# Pharmacovigilance analysis of cutaneous adverse drug reactions for cetuximab, panitumumab, and necitumumab based on EudraVigilance and WHO VigiAccess

**DOI:** 10.3389/fphar.2026.1791359

**Published:** 2026-06-10

**Authors:** Natalia Sauer, Piotr Giedziun, Jacek Calik, Anna Wiela-Hojeńska

**Affiliations:** 1 Department of Clinical Pharmacology, Faculty of Pharmacy, Wroclaw Medical University, Wroclaw, Poland; 2 Department of Artificial Intelligence, Wroclaw University of Science and Technology, Wrocław, Poland; 3 Old Town Clinic, Wrocław, Poland; 4 Division of Histology and Embryology, Department of Human Morphology and Embryology, Faculty of Medicine, Wroclaw Medical University, Wroclaw, Poland

**Keywords:** ADR (adverse drug reaction), EGFR inhibitiors, Eudra Vigilance, pharmacovigilance, VigiAccess database

## Abstract

**Introduction:**

Anti-EGFR monoclonal antibodies (mAbs)--cetuximab, panitumumab, and necitumumab--are cornerstone therapies for selected solid tumors but are frequently associated with cutaneous adverse drug reactions (ADRs). This study aimed to characterize and compare the dermatologic safety profiles of these agents using large-scale post-marketing pharmacovigilance data.

**Methods:**

A comprehensive pharmacovigilance analysis was conducted using aggregated ADR reports from the WHO VigiAccess and European Medicines Agency (EMA) EudraVigilance databases. Disproportionality analyses were performed using reporting odds ratios (RORs) and proportional reporting ratios (PRRs). Dermatologic ADR diversity and similarity were further evaluated using Shannon entropy, principal component analysis (PCA), and multidimensional scaling (MDS).

**Results:**

A total of 50,391, 18,806, and 355 ADR reports were identified for cetuximab, panitumumab, and necitumumab, respectively. Cutaneous ADRs accounted for up to 25% of all reported events. Panitumumab demonstrated the strongest association with skin-related toxicities (ROR = 1.51; PRR = 1.38), particularly acneiform dermatitis, pruritus, and xerosis. Cetuximab exhibited lower relative disproportionality despite higher absolute numbers of reported cases, whereas necitumumab showed a narrower dermatologic toxicity profile, potentially reflecting lower utilization or underreporting. Shannon entropy and dimensionality reduction analyses revealed greater heterogeneity of dermatologic ADRs associated with panitumumab compared with cetuximab and necitumumab. Significant differences in ADR distributions were observed between the WHO and EMA databases.

**Discussion:**

These findings highlight distinct agent-specific dermatologic toxicity signatures among anti-EGFR monoclonal antibodies and demonstrate the value of real-world pharmacovigilance data in complementing clinical trial evidence. Improved recognition of differential toxicity patterns may support personalized monitoring and management strategies for patients receiving EGFR-targeted therapies.

## Introduction

The epidermal growth factor receptor (EGFR, also known as HER1/ErbB1) is a transmembrane receptor tyrosine kinase frequently overexpressed or dysregulated in epithelial malignancies (Zhang, 2023; Wee and Wang, 2017). Ligand-induced EGFR activation initiates intracellular signaling cascades, including the RAS–RAF–MEK–ERK, PI3K–AKT, and JAK–STAT pathways, that drive cellular proliferation, survival, angiogenesis, and migration (Tomuleasa et al., 2024; Lacouture ME. et al., 2011). Given its pivotal role in tumor biology, EGFR has become a major therapeutic target in oncology (Normanno et al., 2006; Sigismund et al., 2018). Monoclonal antibodies (mAbs) targeting the extracellular domain of EGFR represent a clinically validated approach that differs mechanistically from small-molecule tyrosine kinase inhibitors (TKIs), with distinct pharmacodynamics and toxicity profiles (Fauvel and Yasri, 2014; Martinelli et al., 2009).

Three anti-EGFR monoclonal antibodies - cetuximab, panitumumab, and necitumumab have received regulatory approval for use in solid tumors. Cetuximab, a chimeric IgG1, and panitumumab, a fully human IgG2, are widely used in the treatment of metastatic colorectal cancer (mCRC) with wild-type RAS status, while necitumumab, a fully human IgG1, is approved for advanced squamous non-small cell lung cancer (NSCLC) (Van Cutsem et al., 2009; Krawczyk and Kowalski, 2014; Thatcher et al., 2015a). By preventing ligand binding (e.g., EGF, TGF-α), they inhibit EGFR dimerization and subsequent receptor activation (Shyam Sunder et al., 2023). This leads to suppression of downstream signaling cascades such as MAPK/ERK, PI3K/AKT, and JAK/STAT, which are involved in tumor cell proliferation, survival, and metastasis. As a result, these antibodies exert antiproliferative and pro-apoptotic effects on EGFR-expressing cancer cells. Cetuximab and necitumumab, both of the IgG1 subclass, can also activate antibody-dependent cellular cytotoxicity (ADCC) by engaging Fcγ receptors on immune effector cells like NK cells. Panitumumab, being an IgG2 antibody, shows minimal ADCC activity due to its lower affinity for Fcγ receptors.

Thus, despite their therapeutic benefits, EGFR-targeting antibodies are associated with a high incidence of cutaneous and mucocutaneous toxicities, which are often dose-limiting (Lacouture et al., 2018; Arora et al., 2017; Hofheinz et al., 2016; Segaert and Van Cutsem, 2005; Lee et al., 2024). As EGFR is physiologically expressed in basal keratinocytes, sebaceous glands, and hair follicles, its inhibition disrupts normal skin homeostasis (Tran et al., 2012). The most common manifestation is a papulopustular acneiform rash, typically appearing on the face, chest, and upper back, affecting over 70%–90% of patients receiving EGFR mAbs (Fabbrocini et al., 2015). Other frequent toxicities include xerosis, pruritus, paronychia, alopecia, and mucositis. Although most reactions are reversible, they can negatively impact quality of life and treatment adherence. Notably, the severity of the rash has been correlated with treatment response in several studies, serving as a potential surrogate biomarker for efficacy (Balagula et al., 2010).

Given the high prevalence and clinical relevance of dermatologic adverse events associated with anti-EGFR monoclonal antibodies, there is a clear need for systematic real-world evaluation of their safety profiles. Although cetuximab, panitumumab, and necitumumab share a common therapeutic target, they differ in molecular structure, immunoglobulin subclass, degree of humanization, Fc-mediated immune activity, and EGFR-binding characteristics. These pharmacologic differences may translate into clinically meaningful variation in dermatologic toxicity profiles that cannot be assumed to be equivalent across agents. Leveraging large-scale pharmacovigilance databases such as the WHO VigiAccess and EMA EudraVigilance systems provides a robust opportunity to identify agent-specific toxicity signatures in real-world settings.

We therefore hypothesized that these structural and pharmacologic differences result in detectable agent-specific dermatologic toxicity signatures in large-scale pharmacovigilance datasets. The primary objective of this study was to compare the reporting frequency and disproportionality of cutaneous adverse drug reactions associated with these three anti-EGFR monoclonal antibodies using the WHO VigiAccess and EMA EudraVigilance pharmacovigilance databases. Secondary exploratory objectives included quantification of dermatologic toxicity diversity using Shannon entropy and characterization of multidimensional toxicity relationships using dimensionality-reduction techniques (PCA and MDS).

## Materials and methods

### Data source and study design

This retrospective pharmacovigilance study was conducted using publicly available data from two international adverse event reporting databases: VigiAccess™, the open-access interface of the World Health Organization’s (WHO) VigiBase®, and the European Medicines Agency’s (EMA) EudraVigilance database.

VigiBase, maintained by the Uppsala Monitoring Centre (UMC), aggregates anonymized individual case safety reports (ICSRs) submitted by over 130 national pharmacovigilance authorities. VigiAccess provides public access to aggregated summaries of reported adverse drug reactions (ADRs), categorized by drug name and MedDRA (Medical Dictionary for Regulatory Activities) terminology.

EudraVigilance is the official pharmacovigilance system of the EMA and contains ICSRs for drugs authorized in the European Economic Area (EEA). By integrating both data sources, the study aimed to ensure global and regional representativeness and allow for cross-validation of pharmacovigilance signals.

### Selection of drugs and data extraction

The analysis focused on three monoclonal antibodies targeting the epidermal growth factor receptor (anti-EGFR mAbs): cetuximab, panitumumab, and necitumumab (Table 1). These agents were selected due to their clinical relevance in EGFR-expressing solid tumors and their differing molecular structures and mechanisms of action. In May 2025, both VigiAccess™ and EudraVigilance databases were queried using the international non-proprietary name (INN) of each antibody.

**TABLE 1:** Overview of three anti-EGFR monoclonal antibodies used in the treatment of solid tumors.

Active ingredient	Antibody type	Brand names	Molecular formula	Target	Main indications	Year of approval
Cetuximab	Chimeric IgG1	Erbitux, Alzucin, Cetubex, Cetuprima, Cetuxan, Emgrast, C-tuximab, Biotuximab	C225 - recombinant mAb	EGFR	Colorectal cancer (KRAS wild-type), head and neck cancer	2004 (EU/US)
Panitumumab	Fully human IgG2	Vectibix, panitumumab Krka, panitumumab Accord, P-Mab, Panimab	Recombinant IgG2κ mAb	EGFR	Metastatic colorectal cancer (KRAS wild-type)	2006 (EU/US)
Necitumumab	Fully human IgG1	Portrazza	Recombinant human mAb	EGFR	Squamous NSCLC	2015 (US/EU)

### Data analysis

Descriptive statistics were used to summarize the frequency and distribution of cutaneous and mucocutaneous adverse drug reactions (ADRs) associated with cetuximab, panitumumab, and necitumumab. For each agent, the total number of individual case safety reports (ICSRs) was recorded, along with the number and proportion of reports categorized under relevant MedDRA System Organ Classes (SOCs), including *Skin and subcutaneous tissue disorders*, *Nail disorders*, *Hair disorders*, and *Mucosal disorders*.

Preferred Terms (PTs) of interest included acneiform rash, xerosis, pruritus, erythema, paronychia, nail dystrophy, alopecia, trichomegaly, stomatitis, oral ulcers, and mucositis. Results were extracted as aggregated data from both VigiAccess™ and EudraVigilance. Given the structure of spontaneous reporting systems, individual-level information (e.g., age, sex, indication, comorbidities) was not available, and duplicate or confounded entries could not be excluded.

To assess disproportionality in ADR reporting, two statistical measures were applied: the Reporting Odds Ratio (ROR) and the Proportional Reporting Ratio (PRR).

The ROR was calculated as:

$$ROR=\frac{\left(a/b\right)}{\left(c/d\right)}$$

where *a* = number of reports for the target ADR, *b* = number of other skin-related ADRs for the same drug, *c* = total number of skin-related ADRs, and *d* = total number of non-skin ADRs. The ROR provides an estimate of disproportionality within the spontaneous reporting data; values > 1 indicate a higher-than-expected frequency of the specific ADR relative to other skin-related events.

A ROR >1 indicates that the ADR is reported more frequently than expected for that agent. The 95% confidence intervals (CI) for ROR were computed using:

$$SE\left(lnROR\right)=\sqrt{\frac{1}{a}}+\sqrt{\frac{1}{b}}+\sqrt{\frac{1}{c}}+\sqrt{\frac{1}{d}}$$

$$95\%CI=e^{lnROR\pm 1.96\times SE\left(lnROR\right)}$$

In parallel, Proportional Reporting Ratio (PRR) was calculated to measure the relative proportion of a given ADR among all reports for one drug compared to that proportion among all other drugs. The formula was:

$$PRR=\frac{a/\left(a+b\right)}{c/\left(c+d\right)}$$

A PRR value greater than 1 suggests a higher-than-expected frequency of the given ADR for the drug of interest relative to the background frequency. Both ROR and PRR were reported along with their 95% confidence intervals (CI), which were calculated using logarithmic transformation and standard error estimation methods.

$$SE\left(lnPRR\right)=\sqrt{\frac{1}{a}}+\sqrt{\frac{1}{a+b}}+\sqrt{\frac{1}{c}}+\sqrt{\frac{1}{c+d}}$$

$$95\%CI=e^{lnPRR\pm 1.96\times SE\left(lnPRR\right)}$$

To define a positive signal, we adopted criteria consistent with previous pharmacovigilance research:

PRR ≥2, ROR ≥2, number of reports (a) ≥ 3, and chi-squared (χ^2^) ≥ 4.

All analyses were conducted using aggregated publicly available data. As with all spontaneous reporting systems, findings are hypothesis-generating and should be interpreted with caution due to known limitations, including underreporting, variable data quality, and absence of denominator information (i.e., number of patients exposed).

### Shannon entropy analysis

To evaluate the distributional complexity of dermatologic adverse event (AE) profiles, Shannon entropy was calculated for each anti-EGFR monoclonal antibody. The analysis was based on the relative frequencies of the 20 most commonly reported skin and subcutaneous tissue-related adverse drug reactions (ADRs) extracted from the VigiAccess™ database.

For each drug, the number of reports per selected ADR was normalized to obtain a probability distribution p_i_ where p_i_ denotes the proportion of reports for ADRs among all dermatologic events associated with that agent. Entropy was then computed using the standard Shannon formula:

$$H=-\sum_{i=1}^{n}p_{i}{log}_{2}p_{i}$$

where *n* represents the total number of ADR categories included (n = 20). ADRs with zero frequency were excluded from logarithmic computation. The resulting entropy values (expressed in bits) quantify the diversity and evenness of reported dermatologic events, with higher values indicating broader heterogeneity in the AE profile.

All entropy calculations were performed using Python v3.11, applying standard numerical libraries for probability normalization and logarithmic transformation.

Disproportionality analysis (ROR, PRR) was selected to test the primary hypothesis that dermatologic adverse event reporting differs between anti-EGFR antibodies beyond expected background variability. Shannon entropy analysis was applied as an exploratory metric to quantify differences in dermatologic toxicity diversity between agents, while PCA and MDS were used to evaluate whether multidimensional toxicity profiles cluster according to antibody-specific characteristics.

### Dimensionality reduction analysis

To explore similarities and differences in dermatologic adverse drug reaction (ADR) profiles across anti-EGFR monoclonal antibodies, dimensionality-reduction analyses were performed using Principal Component Analysis (PCA) and Multidimensional Scaling (MDS). These methods were applied to normalized reporting frequencies of the most commonly reported skin-related ADRs to visualize multidimensional relationships between agents. PCA was used to identify principal directions of variance in ADR reporting patterns, whereas MDS was applied to represent pairwise dissimilarities between drugs in a low-dimensional space while preserving distance structure. All analyses were conducted using Python (version 3.11) with standard scientific computing libraries.

### Clinical data

Clinical images and case descriptions presented in Figure 6 were derived from retrospective archival data collected at the Old Town Clinic (Wrocław, Poland) between 2017 and 2025 during routine standard-of-care oncology treatment. The dataset included 55 patients treated with anti-EGFR monoclonal antibodies, all with documented and clinically verified courses of cutaneous adverse drug reactions (ADRs).

Dermatologic manifestations were recorded as part of routine clinical practice and included photographic documentation, dermatoscopy, and detailed clinical assessment performed by treating physicians. No additional diagnostic or research-related procedures were introduced for the purposes of this study.

All patients had provided written informed consent for clinical photography and for the use of anonymized medical data and images for scientific and educational publication purposes. All images were fully anonymized prior to inclusion in the manuscript, and no identifiable information is present. The retrospective use of archival data collected during routine care was conducted in accordance with the Declaration of Helsinki and did not require additional ethics committee approval under applicable institutional regulations.

From this cohort, the most representative cases were selected to illustrate the typical clinical spectrum and severity of anti-EGFR–associated skin toxicities. The presented images are intended for illustrative purposes and do not represent the full distribution of observed reactions.

## Results

A total of 50,391 adverse drug reaction (ADR) reports were recorded for cetuximab, 18,806 for panitumumab, and 355 for necitumumab. Comparative analysis revealed statistically significant differences in patient sex, age group, and geographic origin across the three agents.

Sex distribution differed significantly among the drugs, χ^2^(4, N = 69,552) = 218.70, *p* < .001 (Bonferroni-adjusted), with a small effect size (Cramér’s V = .04), with necitumumab showing the highest proportion of male patients (77%), compared to cetuximab (63%) and panitumumab (57%). Age distribution was also significantly associated with the administered drug, χ^2^(10, N = 69,552) = 119.47, *p* < .001, V = .03. Notably, reports involving patients aged ≥65 years comprised 63% of necitumumab-related cases, compared to 33% for cetuximab and 49% for panitumumab. Furthermore, geographic distribution varied significantly across drugs, χ^2^(8, N = 69,552) = 2775.14, *p* < .001, V = .14. While cetuximab and panitumumab reports were primarily from the Americas and Europe, the majority of necitumumab cases (52%) originated from Asia.

**FIGURE 1:**
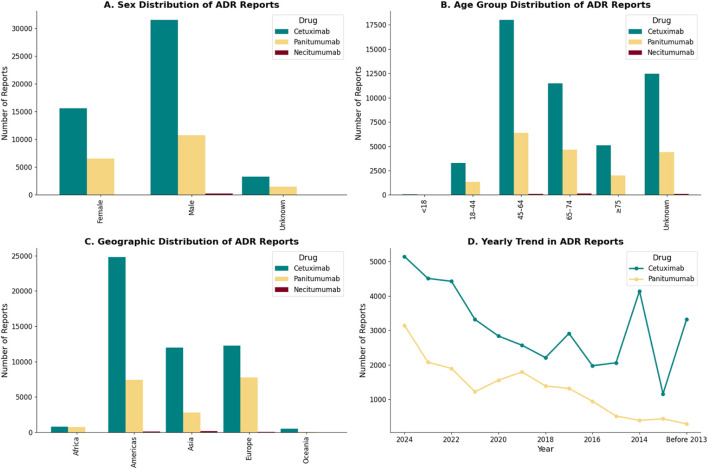
Demographic and geographic characteristics of adverse drug reaction (ADR) reports for cetuximab, panitumumab, and necitumumab. **(A)** Sex distribution of patients with reported ADRs. **(B)** Age group distribution of ADR reports across agents. **(C)** Geographic distribution of ADRs by continent. **(D)** Temporal trends in ADR reports between 2013 and 2025 for cetuximab and panitumumab. Bar colors represent cetuximab (turquoise), panitumumab (buttery yellow), and necitumumab (burgundy). Chi-square tests confirmed significant differences across all comparisons (sex, age, and geography; *p* < 0.0001).

These findings suggest notable differences in ADR reporting patterns for anti-EGFR agents, potentially influenced by variations in patient populations, regional drug availability, and reporting practices (Figure 1; Supplementary Table 1).

### Distribution of most common SOCs

Adverse drug reactions (ADRs) reported for cetuximab (*n* = 90,920), panitumumab (*n* = 39,014), and necitumumab (*n* = 574) showed distinct distributions across System Organ Class (SOC) categories (Table 2).

**TABLE 2:** Distribution of adverse drug reactions across system organ classes (SOCs) for cetuximab, panitumumab, and necitumumab.

System organ class (SOC)	Cetuximab (n = 90920)	Panitumumab (n = 39014)	Necitumumab (n = 574)
Blood and lymphatic system disorders	3398 (3.7%)	1233 (3.2%)	73 (12.7%)
Cardiac disorders	1976 (2.2%)	367 (0.9%)	22 (3.8%)
Eye disorders	884 (1.0%)	767 (2.0%)	1 (0.2%)
Gastrointestinal disorders	8367 (9.2%)	3626 (9.3%)	36 (6.3%)
General disorders and administration site conditions	12535 (13.8%)	5250 (13.5%)	56 (9.8%)
Hepatobiliary disorders	576 (0.6%)	436 (1.1%)	5 (0.9%)
Infections and infestations	5304 (5.8%)	2882 (7.4%)	57 (9.9%)
Injury, poisoning and procedural complications	10160 (11.2%)	2289 (5.9%)	17 (3.0%)
Investigations	4358 (4.8%)	1565 (4.0%)	76 (13.2%)
Metabolism and nutrition disorders	3298 (3.6%)	1593 (4.1%)	46 (8.0%)
Musculoskeletal and connective tissue disorders	1266 (1.4%)	630 (1.6%)	0 (0.0%)
Neoplasms benign, malignant and unspecified (incl cysts and polyps)	2810 (3.1%)	2073 (5.3%)	12 (2.1%)
Nervous system disorders	3856 (4.2%)	1590 (4.1%)	21 (3.7%)
Psychiatric disorders	816 (0.9%)	492 (1.3%)	2 (0.3%)
Renal and urinary disorders	1002 (1.1%)	409 (1.0%)	10 (1.7%)
Reproductive system and breast disorders	151 (0.2%)	123 (0.3%)	0 (0.0%)
Respiratory, thoracic and mediastinal disorders	5600 (6.2%)	1602 (4.1%)	37 (6.4%)
Skin and subcutaneous tissue disorders	16543 (18.2%)	9801 (25.1%)	67 (11.7%)
Surgical and medical procedures	896 (1.0%)	766 (2.0%)	1 (0.2%)
Vascular disorders	3234 (3.6%)	693 (1.8%)	24 (4.2%)

For all three agents, the most frequently reported SOC was “Skin and subcutaneous tissue disorders”, accounting for 18.2% of ADRs for cetuximab, 25.1% for panitumumab, and 11.7% for necitumumab. “General disorders and administration site conditions” ranked second for cetuximab (13.8%) and panitumumab (13.5%), and third for necitumumab (9.8%).

Notably, necitumumab exhibited a higher relative frequency of “Blood and lymphatic system disorders” (12.7%) and “Investigations” (13.2%) compared to cetuximab (3.7% and 4.8%, respectively) and panitumumab (3.2% and 4.0%). Conversely, cetuximab and panitumumab demonstrated higher proportions of “Injury, poisoning and procedural complications” and “Infections and infestations” than necitumumab.

To assess whether the distribution of adverse drug reactions across System Organ Classes (SOCs) differs significantly among cetuximab, panitumumab, and necitumumab, a chi-square test of independence was performed. The test revealed a highly significant association (χ^2^(38) = 3616.46, p < 0.0001, V = .12), indicating that the SOC profile of ADRs varies substantially depending on the anti-EGFR agent.

**FIGURE 2:**
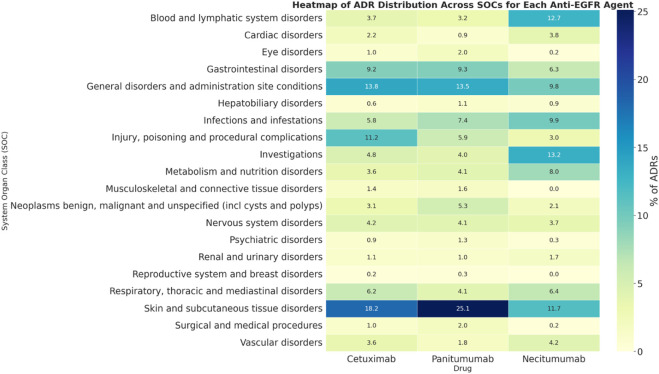
Heatmap of adverse drug reaction (ADR) distributions across System Organ Classes (SOCs) for cetuximab, panitumumab, and necitumumab. Each cell represents the percentage of total ADR reports for a given drug that fall into a specific SOC. Cetuximab (n = 90,920), panitumumab (n = 39,014), and necitumumab (n = 574) display distinct safety profiles.

The distribution of adverse drug reactions (ADRs) across System Organ Classes (SOCs) varied substantially between cetuximab (*n* = 90,920), panitumumab (*n* = 39,014), and necitumumab (*n* = 574), as visualized in Figure 2.

Skin and subcutaneous tissue disorders were the most frequently reported ADR category overall. Panitumumab showed the highest relative frequency (25.1%), followed by cetuximab (18.2%) and necitumumab (11.7%). A pairwise comparison using a Z-test confirmed the difference between panitumumab and cetuximab as statistically significant (*Z* = 28.5, *p* < 0.0001).The absolute difference in proportions was 0.069 (95% CI [0.064, 0.074]).

Necitumumab demonstrated a distinct toxicity profile, with a notably higher proportion of reports related to blood and lymphatic system disorders (12.7%) and investigations (13.2%) compared to cetuximab (3.7% and 4.8%, respectively) and panitumumab (3.2% and 4.0%). The difference in hematologic ADRs between necitumumab and panitumumab was also statistically significant (*Z* = 12.8, *p* < 0.0001), with an absolute difference of 0.096 (95% CI [0.068, 0.123]).

In contrast, cetuximab and panitumumab exhibited higher frequencies of injury-related and infection-related SOCs. For instance, “Injury, poisoning and procedural complications” accounted for 11.2% of cetuximab ADRs *versus* 6.2% for panitumumab and only 3.0% for necitumumab.

A global chi-square test comparing the distribution across all SOCs confirmed significant heterogeneity among the three drugs (χ^2^(38) = 3616.46, *p* < 0.0001). Hierarchical clustering based on the normalized SOC percentages grouped cetuximab and panitumumab closely, whereas necitumumab formed a separate cluster, highlighting its divergent SOC-level toxicity profile.

### Distribution and frequency of skin and subcutaneous tissue-related adverse drug reactions

A total of 16,543, 9,801, and 67 individual case safety reports (ICSRs) related to *skin and subcutaneous tissue disorders* were identified for cetuximab, panitumumab, and necitumumab, respectively (Table 3). These accounted for 18% of all ADRs reported for cetuximab, 25% for panitumumab, and 12% for necitumumab.

**TABLE 3:** Overall, cetuximab and panitumumab demonstrated comparable dermatologic safety profiles, characterized predominantly by rash, acneiform eruptions, and pruritus. In contrast, necitumumab was associated with fewer and less diverse dermatologic reports, possibly due to lower utilization or underreporting.

Cetuximab (n = 16,543)			Panitumumab (n = 9801)			Necitumumab (n = 67)		
Adverse event (MedDRA term)	N (reports)	ADR rate (% of skin ADRs)	Adverse event (MedDRA term)	N (reports)	ADR rate (% of skin ADRs)	Adverse event (MedDRA term)	N (reports)	ADR rate (% of skin ADRs)
Rash	6483	39.2%	Rash	3842	39.2%	Rash	28	41.8%
Pruritus	2512	15.2%	Acne	1341	13.7%	Dermatitis acneiform	18	26.9%
Acne	2133	12.9%	Dry skin	1322	13.5%	Rash maculo-papular	4	6.0%
Dermatitis acneiform	1472	8.9%	Pruritus	1050	10.7%	Dry skin	3	4.5%
Erythema	1353	8.2%	Skin toxicity	895	9.1%	Skin disorder	3	4.5%
Dry skin	1334	8.1%	Erythema	879	9.0%	Erythema	2	3.0%
Urticaria	897	5.4%	Dermatitis acneiform	824	8.4%	Pruritus	2	3.0%
Skin fissures	713	4.3%	Skin fissures	584	6.0%	Skin reaction	2	3.0%
Skin reaction	578	3.5%	Skin exfoliation	503	5.1%	Acne	1	1.5%
Skin toxicity	473	2.9%	Skin reaction	411	4.2%	Acquired perforating dermatosis	1	1.5%
Skin exfoliation	453	2.7%	Blister	374	3.8%	Alopecia	1	1.5%
Hyperhidrosis	344	2.1%	Skin burning sensation	370	3.8%	Dermal cyst	1	1.5%
Alopecia	325	2.0%	Dermatitis	298	3.0%	Dermatitis	1	1.5%
Dermatitis	300	1.8%	Skin disorder	264	2.7%	Hyperhidrosis	1	1.5%
Skin disorder	293	1.8%	Skin lesion	236	2.4%	Hyperkeratosis	1	1.5%
Rash pruritic	252	1.5%	Skin haemorrhage	231	2.4%	Rash erythematous	1	1.5%
Blister	240	1.5%	Alopecia	224	2.3%	Rash pruritic	1	1.5%
Rash maculo-papular	232	1.4%	Pain of skin	211	2.2%	Skin fissures	1	1.5%
Skin burning sensation	228	1.4%	Rash pruritic	182	1.9%	Skin toxicity	1	1.5%
Skin lesion	222	1.3%	Skin irritation	180	1.8%	Toxic skin eruption	1	1.5%

Across all three agents, the most frequently reported dermatologic adverse event was rash, comprising 39.2% (n = 6483) of skin-related ADRs for cetuximab, 39.2% (n = 3842) for panitumumab, and 41.8% (n = 28) for necitumumab.

Other common events included pruritus (15.2% for cetuximab; 10.7% for panitumumab; 3.0% for necitumumab), acne (12.9%, 13.7%, and 1.5%, respectively), and dermatitis acneiform (8.9%, 8.4%, and 26.9%, respectively). While the general dermatologic profiles of cetuximab and panitumumab were largely similar—both showing high rates of acneiform eruptions and dry skin—necitumumab exhibited a narrower ADR spectrum, likely reflecting its lower total number of reported cases.

Dry skin was reported in 8.1% (n = 1334) of cetuximab-related skin ADRs and 13.5% (n = 1322) for panitumumab, but only in 3 cases for necitumumab. Additionally, skin toxicity, skin fissures, and erythema were also frequently reported across all agents, although with much lower absolute numbers for necitumumab.

Disproportionality analysis compared the reporting frequency of skin and subcutaneous tissue disorders among three anti-EGFR monoclonal antibodies: cetuximab, panitumumab, and necitumumab (Table 4). Reporting Odds Ratios (ROR), Proportional Reporting Ratios (PRR), and corresponding 95% confidence intervals (CI) were calculated for each agent in relation to the combined dataset of the remaining two drugs.

**TABLE 4:** Disproportionality analysis of skin and subcutaneous tissue adverse drug reactions (ADRs) reported for cetuximab, panitumumab, and necitumumab.

Drug	Skin ADRs (a)	Non-skin ADRs (b)	Skin ADRs in others (c)	Non-skin ADRs in others (d)	ROR	ROR 95% CI (lower)	ROR 95% CI (upper)	PRR	PRR 95% CI (lower)	PRR 95% CI (upper)	Chi-squared (χ^2^)
Cetuximab	16543	74377	9868	29720	0.67	0.65	0.69	0.73	0.71	0.75	187.33
Panitumumab	9801	29213	16610	74884	1.51	1.47	1.56	1.38	1.36	1.41	459.99
Necitumumab	67	507	26344	103590	0.52	0.4	0.67	0.58	0.35	0.8	20.81

Reporting Odds Ratios (ROR) and Proportional Reporting Ratios (PRR) with 95% confidence intervals (CI) and chi-squared (χ^2^) statistics were calculated by comparing each drug against the others. Values of ROR, and PRR, above 1 indicate a higher-than-expected reporting frequency of skin ADRs, relative to comparators.

Panitumumab demonstrated the highest disproportionality signal for skin-related adverse drug reactions (ADRs), with a ROR of 1.51 (95% CI: 1.47–1.56) and a PRR of 1.38 (95% CI: 1.36–1.41). The chi-squared statistic for panitumumab was χ^2^ = 459.99, well above the conventional threshold of 4, indicating a statistically robust association.

In contrast, cetuximab was associated with a lower-than-expected frequency of skin ADRs, yielding a ROR of 0.67 (95% CI: 0.65–0.69) and a PRR of 0.73 (95% CI: 0.71–0.75), despite a large absolute number of reports (n = 16,543). The chi-squared value (χ^2^ = 187.33) still reflected a significant disproportionality relative to the comparator group.

Necitumumab, with only 67 reports of skin ADRs, showed the weakest disproportionality signal. The calculated ROR was 0.52 (95% CI: 0.40–0.67), and PRR was 0.58 (95% CI: 0.35–0.80), with a chi-squared statistic of χ^2^ = 20.81. Although the number of reports (a ≥3) and chi-squared met signal thresholds, the ROR and PRR did not reach the standard cutoff values (≥2) indicative of a positive signal.

These findings support the observation that panitumumab is more frequently associated with dermatologic toxicity than cetuximab or necitumumab in post-marketing safety reports.

Among cetuximab, panitumumab, and necitumumab, the most frequently reported skin adverse event was rash. Cetuximab showed the highest number of reports (n = 6483), but with a reduced disproportionality signal (ROR = 0.91, 95% CI [0.88, 0.94], χ^2^ = 58.2, p < .001) compared to panitumumab, which had fewer reports (n = 3842) but a significantly elevated ROR (1.47, 95% CI [1.42, 1.52], χ^2^ = 312.5, p < .001). Necitumumab showed a low count (n = 28) and a decreased signal (ROR = 0.42, 95% CI [0.28, 0.63]).

For pruritus, panitumumab again demonstrated a significant disproportionality (n = 1050, ROR = 1.66, 95% CI [1.55, 1.78], χ^2^ = 90.3, p < .001), whereas cetuximab (n = 2512) and necitumumab (n = 2) showed no significant increase (ROR = 0.88, 95% CI [0.83, 0.92]; and 1.25, 95% CI [0.31, 5.00], respectively).

Regarding acne, cetuximab and panitumumab both had substantial counts (n = 2133 and 1341, respectively) with cetuximab showing a mild reduction in disproportionality (ROR = 0.59, 95% CI [0.56, 0.63], χ^2^ = 58.8, p < .001) and panitumumab a moderate increase (ROR = 1.75, 95% CI [1.63, 1.88], χ^2^ = 171.5, p < .001). Necitumumab’s reports were minimal (n = 1), with no reliable signal.

Dermatitis acneiform revealed a markedly elevated disproportionality for panitumumab (n = 824, ROR = 3.12, 95% CI [2.87, 3.40], χ^2^ = 285.6, p < .001), while cetuximab (n = 1472) showed a slight decrease (ROR = 0.87, 95% CI [0.82, 0.92]) and necitumumab (n = 18) had too few reports for conclusive results.

Finally, erythema was moderately elevated for panitumumab (n = 879, ROR = 1.45, 95% CI [1.34, 1.56], χ^2^ = 77.8, p < .001), with cetuximab (n = 1353) showing no significant disproportionality (ROR = 0.94, 95% CI [0.89, 0.99]) and necitumumab again limited by low counts (n = 2).

Overall, panitumumab exhibited stronger disproportionality signals for most common skin adverse events compared to cetuximab and necitumumab, suggesting a higher relative frequency of dermatologic toxicity in post-marketing reports.

**FIGURE 3:**
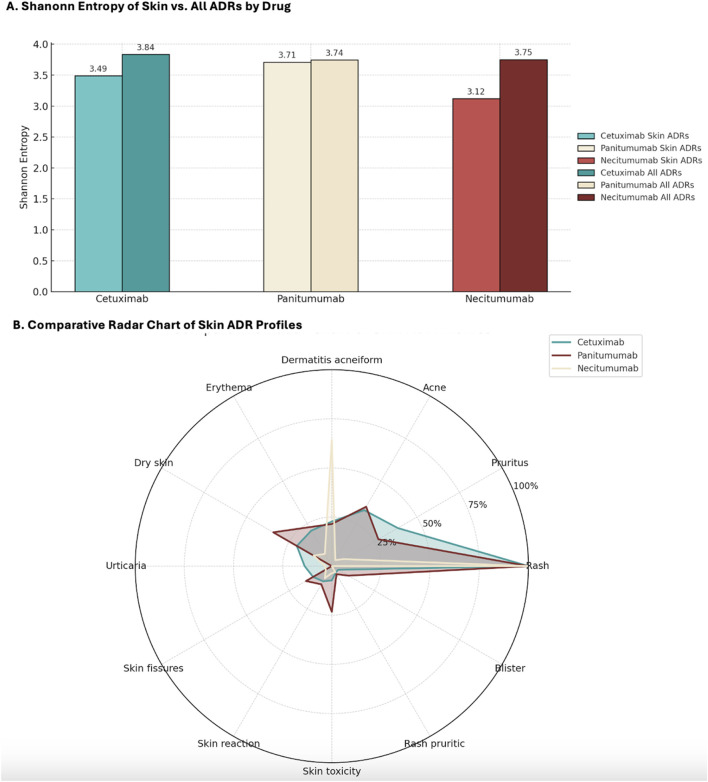
Comparative analysis of adverse drug reaction (ADR) diversity and profiles for cetuximab, panitumumab, and necitumumab. **(A)** Shannon entropy quantifies the heterogeneity of reported skin-related ADRs (light bars) and all ADRs (dark bars) for each drug. Higher entropy values indicate greater diversity of reported adverse reactions. **(B)** A radar chart compares normalized reporting frequencies of the 12 most common skin-related ADRs for each drug, illustrating distinct dermatologic toxicity profiles. Colors correspond to drugs as follows: turquoise (cetuximab), burgundy (panitumumab), and buttery yellow (necitumumab).

Shannon entropy was calculated to assess the diversity of adverse drug reaction (ADR) profiles for cetuximab, panitumumab, and necitumumab, separately for skin-related ADRs and for all reported ADRs (Figure 3A). Entropy values quantify the heterogeneity of reported events, with higher values indicating greater diversity.

For skin-related ADRs, entropy ranged from 3.12 for necitumumab to 3.71 for panitumumab, suggesting that panitumumab has the most heterogeneous dermatologic adverse event profile, while necitumumab’s profile is more limited. Cetuximab showed an intermediate entropy of 3.49. In comparison, the entropy for all ADRs was higher for all drugs, reflecting a broader range of adverse reactions: cetuximab (4.46), panitumumab (4.13), and necitumumab (3.80).

These findings indicate that although panitumumab is associated with a wider variety of skin toxicities, cetuximab and necitumumab demonstrate comparatively narrower dermatologic ADR profiles. The higher overall entropy across all ADRs reflects the complexity of the safety profiles beyond skin reactions.

To explore the patterns of skin-related adverse drug reactions (ADRs) reported for cetuximab, panitumumab, and necitumumab, we conducted dimensionality reduction analyses using Principal Component Analysis (PCA) and Multidimensional Scaling (MDS). These methods allow visualization of complex multivariate data by projecting high-dimensional ADR profiles into a two-dimensional space, thereby revealing similarities and differences between the drugs’ dermatologic toxicity profiles.

PCA identifies directions (principal components) that explain the greatest variance in the data, facilitating interpretation of the dominant patterns. MDS, in contrast, seeks to represent pairwise dissimilarities among observations as distances in a low-dimensional space, preserving the original relationships.

**FIGURE 4:**
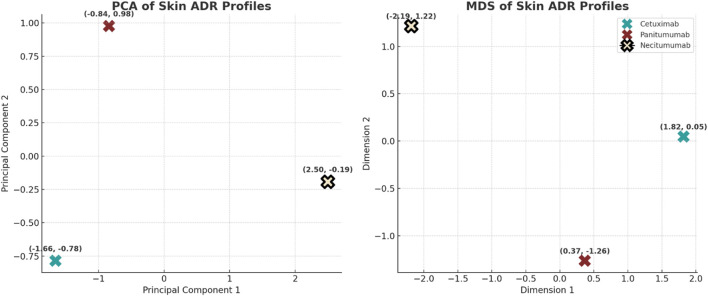
Dimensionality reduction analysis of skin-related adverse drug reaction (ADR) profiles for cetuximab, panitumumab, and necitumumab.

The PCA plot (Figure 4, left panel) demonstrated a clear separation among the three drugs based on their skin ADR profiles. Panitumumab and cetuximab were distinctly separated along the first principal component, reflecting differences in the frequency and diversity of reported skin ADRs. Necitumumab was positioned farther from the other two, consistent with its considerably lower number and variety of reported dermatologic events.

Similarly, the MDS plot (Figure 4, right panel) confirmed the distinct clustering of necitumumab apart from cetuximab and panitumumab, indicating less similarity in its skin ADR profile. Both analyses highlight that necitumumab exhibits a narrower and less frequent spectrum of skin-related toxicities compared to the other agents.

These findings suggest that panitumumab and cetuximab have more complex and heterogeneous skin toxicity profiles, whereas necitumumab’s dermatologic ADR profile is more limited, likely reflecting differences in clinical use, exposure, or reporting.

Left panel (A) shows the Principal Component Analysis (PCA) plot, highlighting the directions of greatest variance in the normalized ADR data. Right panel (B) presents the Multidimensional Scaling (MDS) plot, preserving pairwise dissimilarities between drugs based on their dermatologic toxicity profiles.

Markers represent individual drugs, colored as follows: turquoise for cetuximab, burgundy for panitumumab, and buttery yellow with black border for necitumumab. Numeric labels above markers indicate coordinate values in the respective reduced dimensions.

### Comparative analysis of adverse events: WHO VigiAccess vs. EMA EudraVigilance databases

**FIGURE 5:**
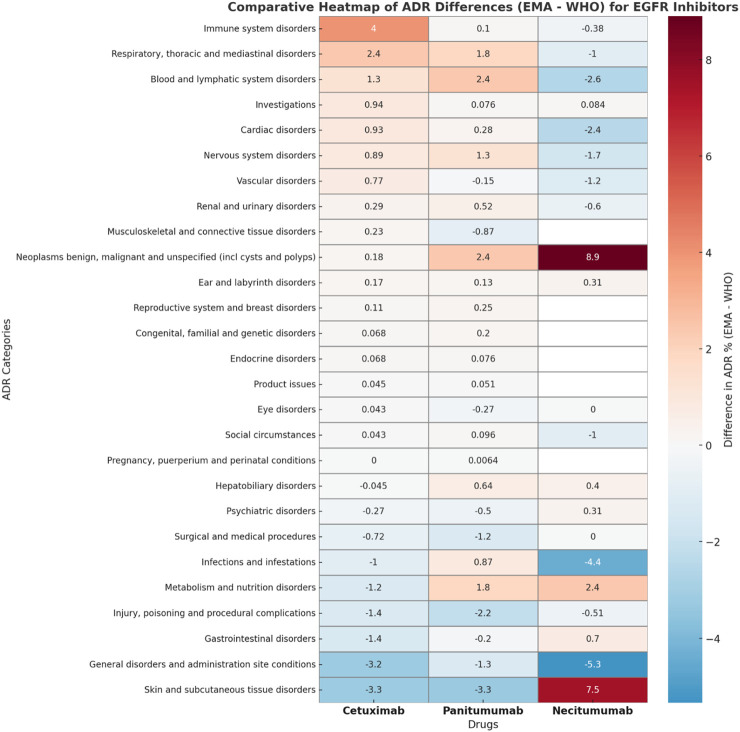
Comparative heatmap showing the differences in percentage of adverse drug reaction (ADR) reports between the European Medicines Agency (EMA) and World Health Organization (WHO) databases for three EGFR inhibitors: cetuximab, panitumumab, and necitumumab. The heatmap illustrates the EMA minus WHO difference in ADR report percentages across various reaction categories. Positive values (shaded in red) indicate a higher percentage of ADR reports in the EMA database, while negative values (blue shading) signify a higher percentage in the WHO database. This visualization highlights areas of concordance and discrepancy in ADR reporting profiles among the three drugs and the two pharmacovigilance sources.

The comparative analysis of adverse drug reaction (ADR) reports for cetuximab, panitumumab, and necitumumab revealed substantial differences in both the volume and distribution of reports between the European Medicines Agency (EMA) and World Health Organization (WHO) pharmacovigilance databases (Figure 5).

Cetuximab had 19,188 ADR reports recorded in the EMA database, while the WHO database documented a substantially larger volume with 90,920 reports. Panitumumab presented 7,493 reports in EMA and 39,014 in WHO. Necitumumab showed the lowest number of ADRs, with 250 reports in EMA and 574 in WHO.

To quantitatively assess whether the distribution of ADR categories differed significantly between the two pharmacovigilance sources, chi-square tests of independence were conducted. These tests evaluate whether the frequency of ADR reports within specific reaction categories is independent of the data source (EMA vs. WHO), thus indicating if reporting patterns vary significantly between databases.

Normalized percentages of ADRs per reaction category were calculated to allow direct comparison. Heatmap visualization of the differences (EMA percentage minus WHO percentage) illustrated heterogeneity in ADR reporting profiles.

Significant differences were identified in key ADR categories across all three drugs. For cetuximab, skin and subcutaneous tissue disorders were significantly more frequently reported in EMA than WHO, χ^2^(1, N = 110,108) = 212.64, p < .001, Cramér’s V = 0.041, indicating a small but meaningful association. Gastrointestinal disorders (χ^2^(1, N = 110,108) = 80.52, p < .001, Cramér’s V = 0.025) and general disorders and administration site conditions (χ^2^(1, N = 110,108) = 196.80, p < .001, Cramér’s V = 0.040) also showed significant differences.

Panitumumab exhibited similar trends with significantly higher reporting of skin disorders in EMA, χ^2^(1, N = 46,507) = 71.22, p < .001, Cramér’s V = 0.036, and general disorders, χ^2^(1, N = 46,507) = 29.85, p < .001, Cramér’s V = 0.023. However, differences in gastrointestinal disorder reports were not statistically significant, χ^2^(1, N = 46,507) = 3.29, p = .070.

For necitumumab, despite lower overall numbers, significant differences were found in skin disorders, χ^2^(1, N = 824) = 13.28, p = .00027, Cramér’s V = 0.104, and general disorders, χ^2^(1, N = 824) = 11.15, p = .0008, Cramér’s V = 0.096. No significant difference was observed for gastrointestinal disorders, χ^2^(1, N = 824) = 0.034, p = .854.

These results indicate notable variability in ADR reporting patterns between EMA and WHO pharmacovigilance databases, particularly for skin-related and general adverse events, emphasizing the value of integrating multiple data sources for comprehensive drug safety evaluations.

### Clinical features of cutaneous adverse reactions associated with anti-EGFR monoclonal antibodies

Beyond the pharmacovigilance data analysis, it is important to consider the clinical presentation of cutaneous adverse reactions associated with anti-EGFR monoclonal antibodies. While the characteristic papulopustular rash remains the most frequently reported dermatologic manifestation, clinicians should also be aware of less commonly emphasized but clinically significant nail disorders.

Nail toxicities, including paronychia, onycholysis, and ingrown nails with associated inflammation, were frequently observed, particularly in patients treated with cetuximab. These nail conditions can lead to pain, secondary infections, and substantial discomfort, potentially affecting patient quality of life and treatment adherence.

The clinical relevance of these nail changes is often underestimated, despite their prevalence. Early recognition and management are crucial to prevent complications.

**FIGURE 6:**
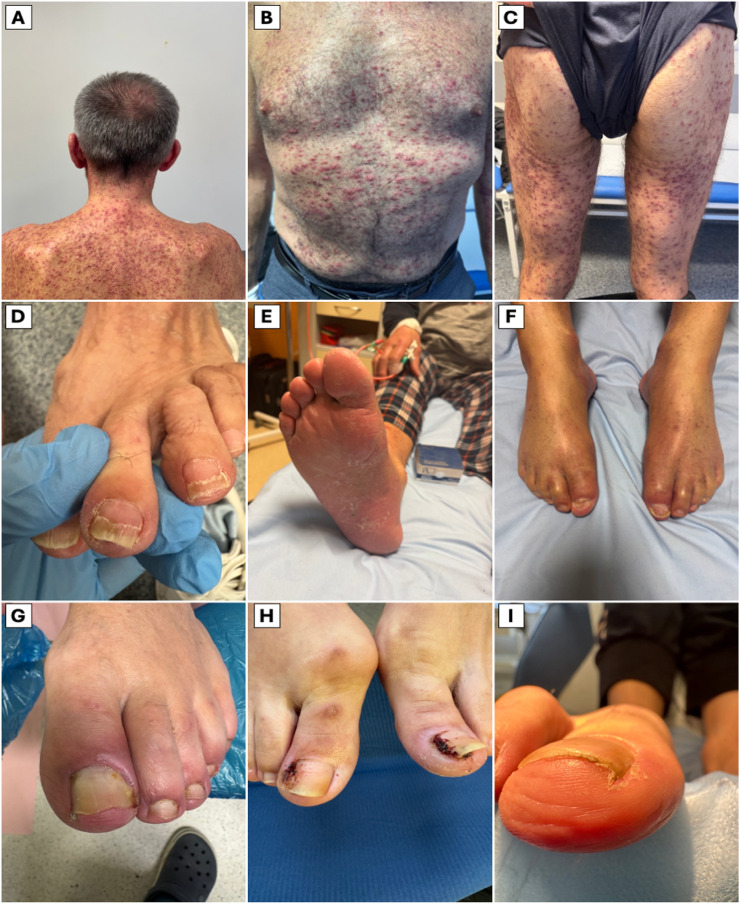
Clinical manifestations of cutaneous adverse reactions associated with anti-EGFR monoclonal antibodies. Panels **(A–D)** illustrate typical skin and nail changes observed in patients treated with panitumumab, including extensive papulopustular rash on the back **(A)**, trunk **(B)**, and thighs **(C)**, as well as nail dystrophy **(D)**.Panels **(E–I)** show cutaneous toxicities related to cetuximab, featuring plantar scaling and erythema **(E)**, toe swelling and deformities consistent with paronychia and onycholysis **(F, G)**, as well as ingrown toenails with associated acute and chronic paronychia and periungual inflammation **(H, I)**.

Representative clinical manifestations are illustrated in Figure 6. Panels A–D show typical papulopustular rash in patients treated with panitumumab, while Panels E–I depict various nail toxicities and inflammatory changes frequently observed with cetuximab therapy.

These clinical features complement the pharmacovigilance findings and underscore the broad spectrum of cutaneous toxicity associated with anti-EGFR therapies.

## Discussion

The cutaneous safety profile of anti-EGFR monoclonal antibodies reflects a complex interplay between molecular structure, immunologic mechanisms, and real-world clinical management (Ji et al., 2025). Our pharmacovigilance analysis confirms that skin toxicities—particularly acneiform rash, dermatitis, and pruritus—remain among the most frequently reported adverse reactions associated with cetuximab, panitumumab, and necitumumab. These events are not merely cosmetic in nature; rather, they exert a substantial impact on treatment adherence, patient quality of life, and overall healthcare utilization (Cury-Martins et al., 2020). Cetuximab and necitumumab are chimeric IgG1 monoclonal antibodies, whereas panitumumab is a fully human IgG2 antibody (Basak et al., 2021). This structural distinction renders cetuximab and necitumumab more prone to immune-mediated effects, including infusion reactions, while panitumumab, owing to its fully human structure, is generally associated with fewer infusion-related events (Cai et al., 2020). Moreover, the IgG1 Fc region of cetuximab and necitumumab enables the induction of antibody-dependent cellular cytotoxicity (ADCC), a mechanism that is considerably less pronounced with the IgG2 subtype of panitumumab (Seo et al., 2014). These differences in isotype and degree of humanization fundamentally contribute to variations in toxicity profiles and immune responses.

The incidence of rash and dermatologic toxicity associated with anti-EGFR monoclonal antibodies has been consistently documented in phase III clinical trials. For cetuximab, a meta-analysis of phase II and III studies reported an overall incidence of all-grade skin rash of 88.2% (95% CI: 84.8%–91.0%), with grade 3–4 toxicity occurring in 11.3% (95% CI: 8.8%–14.3%) of patients (Su et al., 2009). In the Bonner trial, rash developed in 95% of patients within 35 days (Bonner et al., 2010). Similarly, in pivotal colorectal cancer trials (CRYSTAL, EXTREME, OPUS), acneiform rash was observed in 70%–86% of patients, with grade 3–4 skin toxicity ranging from 9% to 18% (Petrelli et al., 2018; ERBITUX, 2024).

Panitumumab likewise demonstrates a substantial burden of dermatologic adverse events. In the ASPECCT trial, skin and subcutaneous tissue toxicities occurred in 35% of patients, comparable to 36% with cetuximab, with grade 3–4 toxicity rates of approximately 14% in both treatment arms (Price et al., 2014). In the PRIME study, skin toxicity of any grade was reported in up to 90% of patients, while observational data indicate rash rates of approximately 75%, with grade 3–4 events occurring in up to 16% of cases (Watanabe et al., 2023; Bouché et al., 2019; Saif and Kim, 2007).

Necitumumab, evaluated in the SQUIRE trial, exhibited lower yet clinically meaningful rates of dermatologic toxicity, with grade 3–4 rash reported in 4% of patients receiving necitumumab in combination with chemotherapy (Thatcher et al., 2015b). Although overall rates were lower than those reported for cetuximab or panitumumab in colorectal cancer populations, rash remained a characteristic and clinically relevant toxicity. Collectively, randomized phase III trials consistently identify rash and skin toxicity as the most frequent and clinically significant adverse events associated with anti-EGFR therapies (Su et al., 2009; Bonner et al., 2010; Petrelli et al., 2018; ERBITUX, 2024; Price et al., 2014; Watanabe et al., 2023; Bouché et al., 2019; Saif and Kim, 2007; Thatcher et al., 2015), thereby providing an essential clinical benchmark for interpreting real-world pharmacovigilance data.

The observed variability in the frequency and severity of cutaneous adverse drug reactions (CADRs) across these agents likely arises from intrinsic differences in immunogenicity and molecular design (Rajagopal et al., 2024). By engaging ADCC, cetuximab may enhance anti-tumor efficacy while simultaneously contributing to a broader dermatologic toxicity spectrum—a phenomenon less evident with panitumumab (García-Foncillas et al., 2019). This immunologic activity represents a double-edged mechanism, supporting therapeutic benefit while predisposing to immune-mediated adverse effects (Basak et al., 2021). Furthermore, necitumumab has been shown to retain high-affinity binding to EGFR variants such as S468R, which are associated with resistance to both cetuximab and panitumumab (Bagchi et al., 2018). This distinct binding profile may influence not only clinical efficacy but also the immunologic microenvironment contributing to CADRs. Notably, early-onset rash has been correlated with improved treatment outcomes, suggesting that dermatologic toxicity may function both as a pharmacodynamic biomarker and a therapeutic challenge (Brinkmeyer and Moore, 2016; Watanabe et al., 2024). These considerations underscore the importance of early recognition and proactive management of CADRs through structured pharmacovigilance systems and multidisciplinary care.

Importantly, the high incidence of dermatologic toxicity observed in pivotal phase III trials provides a critical reference point for contextualizing our real-world findings. In our analysis, 18% of all adverse drug reaction (ADR) reports for cetuximab, 25% for panitumumab, and 12% for necitumumab involved skin and subcutaneous tissue disorders. Although cetuximab generated the highest absolute number of dermatologic ADRs (n = 16,543), panitumumab demonstrated the strongest disproportionality signal (ROR = 1.51; PRR = 1.38), indicating a higher relative reporting frequency of skin toxicity. In contrast, necitumumab exhibited a narrower dermatologic profile, potentially reflecting lower utilization or underreporting. These differences highlight the necessity for agent-specific surveillance and tailored management strategies in clinical practice.

The divergence in dermatologic toxicity profiles among panitumumab, cetuximab, and necitumumab is unlikely to be attributable solely to statistical variation; rather, it reflects fundamental biological distinctions. Differences in molecular structure, EGFR binding characteristics, immune effector function, pharmacokinetics, and dosing schedules contribute to clinically meaningful variability. Cetuximab and necitumumab are IgG1 antibodies, whereas panitumumab is an IgG2 antibody, conferring distinct capacities to engage immune effector mechanisms (García-Foncillas et al., 2019; Trivedi et al., 2016). The IgG1 isotype facilitates ADCC via natural killer cells, macrophages, and neutrophils, and promotes dendritic cell maturation and T-cell priming (Trivedi et al., 2016), whereas IgG2-mediated activity is comparatively limited and primarily involves myeloid lineage cells through FcγRIIa engagement (Rösner et al., 2019; Schneider-Merck et al., 2010). Enhanced immune activation may amplify inflammatory cascades within the skin, and EGFR inhibition has been shown to induce type I interferon responses in keratinocytes, potentially exacerbating rash development (Lulli et al., 2016).

These antibodies also recognize distinct, partially overlapping epitopes within EGFR domain III (García-Foncillas et al., 2019; Voigt et al., 2012; Sickmier et al., 2016). Panitumumab demonstrates higher binding affinity to EGFR than cetuximab (Kd ∼0.12 nM vs. 0.31 nM) (Day et al., 2013), potentially resulting in more sustained EGFR blockade and an increased frequency of on-target toxicities such as severe rash and hypomagnesemia, as observed in ASPECCT (Price et al., 2014). Necitumumab exhibits a unique paratope configuration capable of accommodating EGFR variants resistant to cetuximab and panitumumab (Bagchi et al., 2018). Variations in epitope recognition and downstream signaling inhibition may influence EGFR-mediated regulation of keratinocyte proliferation, differentiation, and barrier integrity (Voigt et al., 2012; Signs, 2005; Robert et al., 2005).

Pharmacokinetic differences further shape toxicity patterns. Cetuximab has a shorter half-life (∼5 days) and is administered weekly, panitumumab has a longer half-life (∼7.5 days) with biweekly dosing, and necitumumab has an intermediate half-life (∼14 days) administered twice per cycle (Long et al., 2017; Yang et al., 2010). Real-world data suggest differences in toxicity onset, with cetuximab-associated skin events appearing earlier and more acutely, whereas panitumumab demonstrates a more gradual toxicity course (Jin et al., 2025; Mao et al., 2025). Hypomagnesemia with necitumumab typically develops after several weeks, consistent with cumulative on-target effects (European Medicines Agency (EMA), 2016), and most patients treated with panitumumab develop rash by the second infusion cycle (Bergman et al., 2014).

Mechanistically, all three agents disrupt EGFR signaling in basal keratinocytes and hair follicles, impair epidermal proliferation and differentiation, compromise barrier function, and induce neutrophilic and lymphocytic infiltration underlying acneiform eruptions (Signs, 2005; Robert et al., 2005; Li et al., 2022). Structural differences may also contribute to infusion-related reactions, which occur more frequently with chimeric antibodies such as cetuximab (Price et al., 2014; Paz-Ares et al., 2015). Meta-analytic evidence further supports quantitatively stronger mucocutaneous toxicity with panitumumab, consistent with its higher affinity and sustained receptor blockade (Mao et al., 2025; Petrelli et al., 2018). Collectively, these mechanistic insights provide biological plausibility for the differential disproportionality signals observed in our study and reinforce the importance of anticipatory, agent-specific toxicity management.

Clinical pharmacists are uniquely positioned to enhance pharmacovigilance in oncology, particularly in the detection and management of adverse drug reactions (ADRs). An Italian observational study conducted at the CRO Aviano National Cancer Institute evaluated pharmacist-led pharmacovigilance in patients receiving ten targeted cancer therapies. Among 154 patients observed between February 2013 and April 2015, the introduction of structured pharmacist involvement—including patient interviews, medical record review, and proactive ADR reporting—resulted in a 124.3% increase in spontaneous ADR reporting compared with the prior system (Fornasier et al., 2018; Kelly et al., 2024). Pharmacist-driven interventions such as patient education, medication review, and follow-up have also been shown to reduce preventable ADRs. A systematic review and meta-analysis of randomized controlled trials demonstrated a 14% reduction in adverse drug events (ADEs) with pharmacist intervention compared to usual care (Tragulpianki et al., 2009), while a meta-analysis in older adults reported a 35% reduction in ADR risk with pharmacist-led strategies, compared to an 8% reduction with other interventions (Gray et al., 2023).

Targeted pharmacist involvement is particularly relevant for dermatologic toxicity. A Japanese review reported that pharmacist-managed prophylaxis—including antibiotics and structured skin care—significantly improved adherence to rash-prevention regimens and reduced the incidence of anti-EGFR-related acneiform rash (Fujii, 2024). Pharmacists play a central role in patient education, reinforcement of prophylactic measures, and early management of emerging rashes, thereby supporting treatment continuity (Lacouture M. E. et al., 2011). The study by Urakawa et al. (2021) further demonstrated that collaboration between hospital and community pharmacists enabled earlier detection of skin symptoms and more rapid intervention in patients receiving oral anticancer agents, resulting in reduced severity of skin adverse effects, improved adherence to supportive care recommendations, and fewer therapy interruptions (Urakawa et al., 2021). Collectively, these findings underscore the value of integrating pharmacists into oncology care teams to enhance ADR detection and improve outcomes.

The clinical consequences of inadequately managed skin ADRs are substantial. Severe rash may progress to infection or debilitating sequelae requiring emergency intervention. A prospective study by Lavan et al. identified ADRs as a major contributor to hospital admissions among oncology patients (Lavan et al., 2019), with over 21% of admissions attributed in whole or in part to ADRs and more than 60% deemed preventable or avoidable. These findings highlight the considerable burden of ADRs on hospital utilization and the imperative for improved preventive strategies.

Beyond clinical harm, ADR-related hospitalizations impose significant economic costs. A European cost analysis estimated the mean cost per ADR-related hospitalization at approximately €5,200 (Laroche et al., 2025), with cumulative national expenditures reaching hundreds of millions annually. Even in outpatient settings, dermatologic ADRs increase healthcare utilization through additional clinic visits, home nursing services, and supportive pharmacotherapy. In aggregate, insufficiently monitored skin toxicities generate substantial economic strain (Eilers et al., 2010).

At the patient level, unmanaged toxicity undermines adherence and compromises therapeutic benefit. Patients experiencing severe rash are more likely to omit doses or discontinue therapy altogether (Yu et al., 2020; Burak et al., 2015). In Poland, pharmacists increasingly contribute to medication adherence and supportive oncology care by providing patient education, managing adverse effects, developing individualized pharmaceutical care plans, and ensuring continuity of care following hospital discharge. Their accessibility and sustained engagement position them as integral partners in optimizing treatment safety and effectiveness (Burak et al., 2015).

### Clinical implications

The present findings suggest several clinically relevant differences in how dermatologic toxicity should be anticipated and monitored across anti-EGFR monoclonal antibodies. For panitumumab, the higher proportion of skin and subcutaneous tissue disorders among all reported ADRs, together with stronger disproportionality signals for rash, pruritus, acne, dermatitis acneiform, and erythema, supports a particularly proactive approach to early dermatologic surveillance and supportive skin care from treatment initiation. In contrast, cetuximab showed a broader pattern of clinically relevant cutaneous manifestations, including pruritus, skin fissures, urticaria, blistering, and periungual toxicity, suggesting that monitoring should extend beyond papulopustular rash alone and include systematic assessment of nail and barrier-related complications. Although necitumumab was associated with fewer reported cutaneous events, this finding should not be interpreted as evidence of lower dermatologic risk, given the markedly smaller reporting volume; rather, it suggests that toxicity assessment in necitumumab-treated patients should remain broad and should not focus exclusively on skin events. Importantly, the higher relative contribution of skin-related ADRs in the EMA dataset compared with the WHO dataset indicates that dermatologic toxicities may be captured differently across reporting environments, underscoring the need for consistent clinical recognition and structured reporting of anti-EGFR-associated skin toxicity. Finally, the significant differences in sex, age, and geographic distribution across the three agents indicate that these safety signals arise from clinically distinct exposure populations, which should be taken into account when interpreting between-drug comparisons.

### Limitations

This study has several limitations that should be considered when interpreting the comparative dermatologic safety profiles observed across anti-EGFR monoclonal antibodies.

The most important limitation is the marked imbalance in reporting volume between agents, particularly the substantially smaller number of total and skin-related ADR reports available for necitumumab compared with cetuximab and panitumumab. Given that necitumumab has a shorter market presence, narrower indication spectrum, and lower cumulative exposure, the reduced diversity of dermatologic events observed for this drug likely reflects differences in utilization rather than a truly narrower toxicity profile. This imbalance also affects the stability of disproportionality estimates and limits the interpretability of clustering and dimensionality-reduction analyses involving necitumumab. Therefore, conclusions regarding the relative distinctiveness of its dermatologic profile should be interpreted cautiously.

A second limitation relates to the intrinsic characteristics of spontaneous pharmacovigilance databases. Reporting frequencies reflect reporting behaviour rather than true incidence, and the absence of exposure denominators prevents estimation of absolute dermatologic toxicity risk. As a result, the differences observed between cetuximab and panitumumab should be interpreted as differences in reporting patterns rather than direct measures of comparative clinical toxicity.

Differences identified between the WHO VigiAccess and EMA EudraVigilance datasets also likely reflect regional variation in pharmacovigilance practice and reporting intensity. For example, the higher proportion of skin-related reports in the EMA dataset compared with the WHO dataset suggests that dermatologic toxicities may be more consistently recognized or reported within European oncology settings. Although this variability limits strict cross-database comparability, the overall consistency of dermatologic signal structure across both systems strengthens confidence in the observed comparative patterns.

Another important limitation is the absence of patient-level clinical information, including cancer type, treatment line, prior therapies, supportive care strategies, and concomitant medications. These factors are particularly relevant in the context of anti-EGFR-associated skin toxicity, which is strongly influenced by prophylactic dermatologic management and treatment intensity. Their absence restricts the ability to determine whether differences between agents reflect intrinsic pharmacologic properties or differences in clinical context.

Finally, exploratory analyses based on Shannon entropy and dimensionality-reduction techniques (PCA and MDS) were intended to support interpretation of multidimensional relationships between dermatologic ADR profiles rather than to provide confirmatory statistical inference. These approaches are sensitive to differences in reporting volume and therefore should be interpreted primarily as tools for pattern visualization rather than evidence of definitive biological separation between toxicity profiles.

Taken together, these limitations mean that the present findings should be interpreted as comparative pharmacovigilance signals rather than estimates of true incidence or causal risk. Nevertheless, the consistency of dermatologic reporting patterns across two independent international pharmacovigilance systems and the concordance between descriptive and multidimensional analyses support the robustness of the observed similarities between cetuximab and panitumumab and the cautious interpretation of the apparently narrower dermatologic signal observed for necitumumab.

## Conclusion

In summary, our analysis reinforces that cutaneous ADRs to anti‐EGFR antibodies are common, consequential, and under-recognized. Routine pharmacovigilance - especially when augmented by clinical pharmacists - is critical to identify these events early and manage them proactively. Neglecting skin toxicity not only harms patients (through complications and reduced quality of life) but also burdens the healthcare system (through extra costs and hospitalizations) and ultimately can compromise oncologic outcomes. As leading authorities have concluded, enhancing pharmacovigilance in oncology must be highlighted with every effort to ensure patient safety and quality of life. Our findings make it clear that attentive monitoring of dermatologic ADRs should be a standard component of targeted cancer therapy.

## Data Availability

The original contributions presented in the study are included in the article/Supplementary Material, further inquiries can be directed to the corresponding author.
