# Supplementary material for: Pharmacovigilance analysis of cutaneous adverse drug reactions for cetuximab, panitumumab, and necitumumab based on EudraVigilance and WHO VigiAccess
Source: Front Pharmacol. 2026 Jun 10;17:1791359. doi: 10.3389/fphar.2026.1791359 (PMC13291479; doi:10.3389/fphar.2026.1791359)
Supplement: Supplementary file 1 [file Table1.docx]

**Supplementary Table 1.** Demographic and geographic characteristics of ADR reports for cetuximab, panitumumab, and necitumumab. Values represent absolute numbers with corresponding percentages in parentheses. Statistical comparisons were conducted using chi-square tests for independence: sex, χ²(4) = 218.70, *p* < .001; age group, χ²(10) = 119.47, *p* < .001; and geographic region, χ²(8) = 2775.14, *p* < .001.

| **Parameter** | **Cetuximab** | **Panitumumab** | **Necitumumab** |
| --- | --- | --- | --- |
| **Number of ADR reports** | 50391 (100%) | 18806 (100%) | 355 (100%) |
| **Female** | 15586 (31%) | 6571 (35%) | 63 (18%) |
| **Male** | 31535 (63%) | 10740 (57%) | 275 (77%) |
| **Unknown** | 3270 (6%) | 1495 (8%) | 17 (5%) |
| **0-27 days** | 5 (0%) | 0 (0%) | 0 (0%) |
| **28 days to 23 months** | 6 (0%) | 8 (0%) | 0 (0%) |
| **2-11 years** | 18 (0%) | 6 (0%) | 0 (0%) |
| **12-17 years** | 25 (0%) | 8 (0%) | 0 (0%) |
| **< 18 years** | 54 (0%) | 22 (0%) | 0 (0%) |
| **18-44 years** | 3293 (7%) | 1359 (7%) | 5 (1%) |
| **45-64 years** | 18009 (36%) | 6383 (34%) | 92 (26%) |
| **65-74 years** | 11458 (23%) | 4665 (25%) | 132 (37%) |
| **≥ 75 years** | 5097 (10%) | 1985 (11%) | 26 (7%) |
| **Unknown age** | 12480 (25%) | 4392 (23%) | 100 (28%) |
| **Africa** | 778 (2%) | 762 (4%) | 0 (0%) |
| **Americas** | 24846 (49%) | 7438 (40%) | 130 (37%) |
| **Asia** | 11979 (24%) | 2796 (15%) | 186 (52%) |
| **Europe** | 12285 (24%) | 7732 (41%) | 38 (11%) |
| **Oceania** | 503 (1%) | 78 (0%) | 1 (0%) |
| **2025** | 1656 (3%) | 935 (5%) | 0 (0%) |
| **2024** | 5148 (10%) | 3147 (17%) | 151 (43%) |
| **2023** | 4508 (9%) | 2073 (11%) | 18 (5%) |
| **2022** | 4425 (9%) | 1895 (10%) | 23 (6%) |
| **2021** | 3315 (7%) | 1217 (6%) | 10 (3%) |
| **2020** | 2834 (6%) | 1553 (8%) | 12 (3%) |
| **2019** | 2567 (5%) | 1791 (10%) | 7 (2%) |
| **2018** | 2207 (4%) | 1386 (7%) | 10 (3%) |
| **2017** | 2910 (6%) | 1314 (7%) | 36 (10%) |
| **2016** | 1972 (4%) | 941 (5%) | 23 (6%) |
| **2015** | 2058 (4%) | 513 (3%) | 2 (1%) |
| **2014** | 4142 (8%) | 389 (2%) | 12 (3%) |
| **2013** | 1158 (2%) | 433 (2%) | 4 (1%) |
| **Before 2013** | 3312 (7%) | 285 (2%) | 37 (10%) |

##### 
